# Commercial Devices-Based System Designed to Improve the Treatment Adherence of Hypertensive Patients †

**DOI:** 10.3390/s19204539

**Published:** 2019-10-18

**Authors:** Vandermi João da Silva, Vinicius da Silva Souza, Robson Guimarães da Cruz, Juliana Mesquita Vidal Martínez de Lucena, Nasser Jazdi, Vicente Ferreira de Lucena Junior

**Affiliations:** 1 Institute of Computing, Federal University of Amazonas, UFAM-PPGI, Manaus–Amazonas 69067-005, Brazilsouzavinicius1993@gmail.com (V.d.S.S.); 2 Department of Electronics and Telecommunications, Federal University of Amazonas, UFAM-PPGEE, Manaus–Amazonas 69067-005, Brazil; robsoncruz.ufam@gmail.com; 3 Federal Institute of Education, Science and Technology of Amazonas, IFAM-CMC, Manaus–Amazonas 69067-005, Brazil; juliana.lucena@ifam.edu.br; 4 Institute of Industrial Automation and Software Systems, The University of Stuttgart, 70174 Stuttgart, Germany; Nasser.Jazdi@ias.uni-stuttgart.de; 5 Prof. Nilmar Lins Pimenta Building, Sector North of UFAM’s Main Campus, Technology College, Federal University of Amazonas, UFAM-CETELI, Manaus–Amazonas 69077-00, Brazil

**Keywords:** assisted living, health information management, ambient intelligence, smart homes, medical treatment, medical expert systems

## Abstract

This paper presents an intelligent system designed to increase the treatment adherence of hypertensive patients. The architecture was developed to allow communication among patients, physicians, and families to determine each patient’s medication intake and self-monitoring of blood pressure rates. Concerning the medication schedule, the system is designed to follow a predefined prescription, adapting itself to undesired events, such as mistakenly taking medication or forgetting to take medication on time. When covering the blood pressure measurement, it incorporates best medical practices, registering the actual values in recommended frequency and form, trying to avoid the known “white-coat effect.” We assume that taking medicine precisely and measuring blood pressure correctly may lead to good adherence to the treatment. The system uses commercial consumer electronic devices and can be replicated in any home equipped with a standard personal computer and Internet access. The resulting architecture has four layers. The first is responsible for adding electronic devices that typically exist in today’s homes to the system. The second is a preprocessing layer that filters the data generated from the patient’s behavior. The third is a reasoning layer that decides how to act based on the patient’s activities observed. Finally, the fourth layer creates messages that should drive the reactions of all involved actors. The reasoning layer takes into consideration the patient’s schedule and medication-taking activity data and uses implicit algorithms based on the J48, RepTree, and RandomTree decision tree models to infer the adherence. The algorithms were first adjusted using one academic machine learning and data mining tool. The system communicates with users through smartphones (anytime and anywhere) and smart TVs (in the patient’s home) by using the 3G/4G and WiFi infrastructure. It interacts automatically through social networks with doctors and relatives when changes or mistakes in medication intake and blood pressure mean values are detected. By associating the blood pressure data with the history of medication intake, our system can indicate the treatment adherence and help patients to achieve better treatment results. Comparisons with similar research were made, highlighting our findings.

## 1. Introduction

Context-aware data can be used by modern applications by taking into consideration data about users, such as their locations and their activities, making it possible to infer their daily behavior. An intelligent system may provide customized services based on this information [1]. This concept involves a set of emerging information technologies, such as those present in modern consumer electronics devices, which are increasingly prevalent in peoples’ daily lives and represent the consolidation of pervasive and ubiquitous systems now connected on the cloud. The so-called Internet of Things (IoT) technology allows any appliance to receive an Internet Protocol (IP) address, and thus communicate through the web, allowing remote and local device control [2]. IoT and intelligent environments are even more present in home-automation systems. Additionally, these connected environments can use today’s network technologies to access public services, such as automatic messaging, making them available to diverse applications.

In a connected, intelligent environment, the collected data, such as users’ locations, actions, behavior, and interactions with objects around them, can be stored periodically in a database. Afterward, these data may be analyzed and processed, becoming relevant information that can be used to identify appropriate services made available to the users anywhere. In fact, with the advent of cloud computing and software as a service (SaaS), more services will be offered on the Web [3].

Moreover, services based on data coming from multiple sources are often challenging to manage and validate, which leads to inconsistencies and incorrect or incomplete information. We claim that it is necessary to build a system that concentrates and retrieves data using a contextual dataset with a set of rules and decision-based algorithms to minimize this problem. This paper discusses such a new approach that allows interaction among residential automation systems based on a contextual information model that collects information through sensors in residences and applies decision tree algorithms to establish relevant services with a specific focus on healthcare systems.

The proposed system focuses on treatment adherence for hypertensive patients. The aim is to generate services based on prescription data inputs and contextual data, such as periodic blood pressure measurements, indoor and outdoor patients’ locations, and personal activities. The system can collect these contextual data coming from several devices, store these data in a training base, and generate services based on rules and decision tree algorithms. These algorithms are implemented at one specific layer responsible for the computation of the adherence. The goal is to use the proposed system for the treatment of senior patients who are more likely to forget prescribed times and mistake medications.

The usage of modern digital technologies by elderly individuals may be an impeditive factor for the proper acceptance of smart devices in electronic healthcare solutions. Recent studies discussed this topic, and among the related issues, we highlight that elderly users see no additional value in a new, intrusive technology that aims to help them do something that they have become accustomed to doing for their whole life [4]. Why should they trust or become dependent on those new gadgets? Moreover, the proposed system should not be impersonal, and it is necessary to guarantee the expert medical advice to increase trust; additionally, the cost should not be impeditive such that it influences the elderly patients’ acceptance of the new technological solution [5].

Our system was designed to overcome these concerns. Most of the computational task is done ubiquitously; the computer (i.e., sensors and actuators) is there, but users do not need to perceive its presence. Regarding the human–machine interface, our primary communication mechanism between users and the system is the old and famous TV set. In almost every culture, the TV plays an important role in older adults’ lives, and they watch TV frequently. Additionally, the other communication methods are based on even more popular applications, such as WhatsApp, an even more utilized tool among our users’ group [6]. More technologically sophisticated communication over social media, for example, is primarily focused on relatives and professional caregivers.

One characteristic imposed on the proposed system was that it should make use of commercial devices. The resulting proposal was based on a set of off-the-shelf sensors, consumer electronic devices, and a modified blood pressure meter that arranged together, manage contextual knowledge and try to guarantee adherence to a specific medical treatment. The only exception among these devices is one intelligent medicine cabinet that was developed for medication control purposes and that is not currently commercialized.

To evaluate the results obtained, we selected similar works already published and analyzed their characteristics, covering relevant technological and social issues of each one. Based on this result, we produced a table containing a comparison of our work with the others’ and summarized the unique characteristics of our approach.

This article is an extended and modified version of a paper presented in the 8th International Conference on Current and Future Trends of Information and Communication Technologies in Healthcare (ICTH 2018) [7]. This previous paper focused more on the decision tree algorithms, but here we describe the whole system in detail. The rest of this paper is organized as follows. In the next section, a review of prior works covering related research is presented. In Section 3, we show the concept of the proposed system. Section 4 contains details of the implementation of the prototype. In Section 5, we present the tests performed to verify the correct functioning of our system, the results achieved with the reasoning module, and a detailed comparison with the most similar related works. Finally, the Conclusion section closes the paper and suggests future work.

## 2. Literature Review

In this section, we will present related work for a better understanding of the issues involving therapy adherence and the solutions proposed concerning some computational intelligence to improve patient monitoring. The use of blood pressure measurements as one of the critical parameters for hypertensive patients’ prognosis and the available technologies are described. Based on the literature search, we present some existing solutions, our concerns about the theme, and point out the approach implemented in our work.

The use of wearable health devices (WHDs) and implantable sensors is a current research topic with many usages in the modern way of living [8]. Commercial devices can be found for a wide range of applications, from general fitness accessories to specific cardiovascular monitors. The migration from experimental laboratories to everyday usage through off-the-shelf consumer devices is a growing trend that has led to concerns about patient preferences at an early stage in the design process of these devices [9].

Following the tendency of using commercial devices, the technical performance of medical wireless personal area networks (WPANs) based on smartphones was studied at the University of Málaga [10]. A prototype of a health telemonitoring system that incorporates a commercial Android smartphone acting as a gateway between a set of wireless medical sensors and a data server was described. The authors concluded that present commercial mobile phones are capable enough to combine their regular operation, including multimedia demands, and simultaneously perform as medical monitors or gateways in an mHealth (mobile health) WPAN. These conclusions are the same as those presented by Gu and his coauthors [11] in a work that covered the privacy aspects of the usage of Android-based devices as providers of telemedicine infrastructure.

Most of the studies were targeted to senior patients and the treatments related to common diseases for this group [12,13]. A promising solution points to smart homes that incorporate environmental and wearable medical sensors, actuators, and modern communication and information technologies [14]. The goal of those homes is to allow elderly individuals to stay in their comfortable home environments instead of expensive and limited healthcare facilities. When searching for specific diseases, we found that medication adherence is an issue for almost every long-term treatment. Related research covers heart and cardiovascular disease [15,16], diabetes [17], asthma [18], and other respiratory diseases [19].

According to the World Health Organization (WHO), the adherence to long-term therapy means the extent to which a person makes changes in lifestyle following the recommendations of the healthcare team. These changes include taking the proper dose of medication at the right time and in the right way, dietary adaptations, improvement of physical activities, and others. WHO also emphasizes that adherence implies that the patient agrees with the treatment plan and does not merely comply with it [20]. The latter could mean that the patient comprehends the instructions to a certain level but does not necessarily commit to them, accounting for one of the reasons that contributes yearly, to a vast number of preventable deaths [21].

The usefulness of healthcare services is profoundly affected by treatment adherence, and it may be difficult for patients undertaking the prescribed therapy [22]. Clinical trials show that effective long-term interventions address multiple factors. Among key factors are new methods and modern technological applications for simplifying regimens. Medication adherence is a crucial issue because failing to meet timing, dosage, and frequency requirements can lead to many health drawbacks [23]. Patients can find a large number of commercial solutions that dispense pills on the Internet; one disadvantage of those systems is the necessity to fill them with medicine prior to use.

The main issue is that many factors contribute to abandoning medical treatment, such as unpleasant side effects [24], a lack of involvement from relatives and caregivers [25], and forgetfulness [12]. Many patients, especially senior patients who take multiple drugs for a long-life treatment, cannot remember when, or even if they took their medicine. Patients who take medication after the scheduled time, risk an overdose if they double the doses at the next scheduled time. There are two different approaches to solve this problem. The first is a therapeutic approach, and the second is remote monitoring [26]. There is a high number of reports published in journals and conference proceedings that affirm that medication adherence is still a problem to solve. Most of them assert that the complexity and the challenges involved in this important topic may be addressed by the extensive use of emerging information and computational technologies [21]. Moreover, it is crucial to focus on the usability and acceptability of these electronic devices attempting to fulfill the demands of patients and medical crews [27]. 

Patient monitoring is an essential tool for improving treatment adherence and informing health professionals about patient behavior. That means that a set of sensors must be used to capture the patient’s status and to infer if the medication is not taken correctly, leading to automatic or human intervention. That is not an easy task as, on the one hand, there are not enough caregivers available; on the other hand, systems that make use of expensive hardware and software components are not available everywhere. Additionally, the accuracy of these systems is not yet proven to reach certain levels [28]. 

A system named multimedia healthcare system (MHS) was proposed by a group of researchers from the Northwestern Polytechnical University in Shaanxi [13]. The solution provides some healthcare services, such as an online medication-taking plan, access to the medication diary, recognition of medical information, and advice on effective medication. Additionally, MHS can offer effective, context-aware prompting, based on an understating of the patient’s situation. The architecture proposed for the MHS is a service-based solution consisting of five collaborating services: reminder, multimedicine management, interruption detection, mediation, and presentation service. The system makes use of television sets to communicate with patients and an electronic cabinet to dispense medicine. No feedback on the adherence was related. 

Varshney [12] presents a smart medication management system (SMMS), developed at Georgia State University, that can remotely monitor and regulate patients’ prescriptions. It was designed to support communication with caregivers to improve scheduling. The system’s functionality depends on several sensors spread across a smart home infrastructure. There is also no mechanism that guarantees that the medication is taken accordingly, and the motivation depends strongly on the caregiver personnel. 

Researchers developed an electronic medication management assistant (eMMA) from the Bern University of Applied Sciences [29]. The objective of this approach is coaching patients concerning their medication, reducing medication misuse. Additionally, they proposed algorithms to avoid contraindications and medication errors. The solution is based on a smartphone application, and its functionality relies strongly on Internet availability. Moreover, the attending doctors should agree to share information with the app.

One open question is how to ensure that the prescribed medicine is taken. Electronic mechanisms to alert the right time and the right medicine are prevalent, as seen in the already mentioned examples, but warning the patients does not mean that they will act accordingly. That is why some researchers proposed diverse approaches to detect the act of taking medicine. For example, a smartwatch-based system capable of detecting several body motions was developed by Kalantarian’s group [30]. They claimed that medication adherence may be predicted by using built-in triaxial accelerometers and gyroscopes and that the efficacy of the technique was confirmed through a survey of medication ingestion habits and experimental results on movement classification. 

More invasive solutions make use of ingestible sensors [31,32]. A similar approach was proposed by the researchers at Proteus Digital Health, Inc. Their system detects when medications have been ingested [33,34] by using an ingestible sensor and an external monitor. These two devices can be used to confirm whether, when, and how many doses of prescribed medication are taken. This information is provided wirelessly in a confidential way to patients and health personnel. While this is an exact solution, as the sensor is combined with the medicine, it is also expensive and likely not viable for the average patient. 

Real-time data collection is another commonly addressed issue [35]. The literature relates solutions for controlled ambient settings (hospitals, clinics, and senior homes), as well as for noncontrolled ones (smart homes) [36,37]. Another question relates to what should be done with the collected data. For example, one machine learning algorithm was developed to track wrist motions in real time and identify medication intake activities. The authors proposed a data analysis pipeline to detect medication adherence by examining single-wrist movements reliably [38]. The system achieves an accuracy of 78.3% in adherence detection without the need for pillboxes and with only one sensor worn on either wrist. According to the authors, the accuracy of the algorithm is only 7.9% lower than that of a system with two sensors that tracks the motion of both wrists. 

Another approach presented a machine learning technique for monitoring social media to identify prescription drug abuse [39]. The authors manually wrote down 300 tweets indicating the abuse of illegal drugs. The experiment compared a set of parsers with the J48 and support vector machine (SVM) decision tree algorithm to determine which tweets contained signs of actual drug abuse. After data processing with the algorithms, the J48 algorithm presented an accuracy of 74.8% for the classification of drug abuse and non-abuse.

Chandrasekar and colleagues have presented a method to improve the accuracy of decision tree mining with data preprocessing [40]. They applied a supervised filter to discrete data and used the J48 algorithm to construct a decision tree. The results were compared to those of the J48 without a discrete data filter. Experimental results showed that the accuracy of J48 after discretization is better than that before discretization. The authors used a leukemia dataset with the Fayyad–Irani discretization method implemented in WEKA [41].

Concerning hypertension, not only medication intake but also blood pressure (BP) monitoring can be essential to treatment success. Recent studies indicated that a “white-coat” effect induces higher BP levels when the measurement is taken under supervision at a clinical facility [42,43]. Conversely, home BP monitoring (HBPM) could be more representative of daily life if it is conducted based on at least three days (but, preferably for 6–7 days) consecutively, twice a day, and using a validated BP monitor. If the patient follows appropriate counseling about how and when to take the measurements before each clinical visit, the data of HBPM could be of great value for the prognosis [44]. There is evidence that this kind of patient approach can benefit BP control and is directly related to medication adherence [45,46]. Moreover, HBPM can be a better indicator of target-organ damage [47] and serves as a predictor of cardiovascular disease and mortality [48].

To achieve better results in BP control, not only is medication adherence relevant but also the monitoring of the BP itself [49]. Assuming that there is a positive impact on patients’ health when they actively participate in their treatment [45,46], the European Society of Cardiology (ESC) emphasizes that technological applications are welcome to remind the patients to take BP measurements and store and transmit the data to their medical caretakers [50].

Several devices and systems have been proposed to monitor different body and health parameters, including BP, pulse rhythm, glucose, body weight, body mass index, physical activity, and so on [51,52]. Many of them, as smartphone-based devices, were developed to improve patient adherence and can share the data with persons of interest via the Web [53]. Regarding HBPM, only those based on cuff-measurements are considered to ensure more precise BP data [54], with preferable measures taken on the arm rather than wrist [50]. Table 1 provides an overview of different systems and devices developed for the patient’s home health monitoring.

Many groups are searching for precise methods to measure the actual BP of patients in diverse types of treatment. It is known that body position can affect the values obtained, and techniques were developed to overcome the related effects [55,56,57]. When dealing with clinical situations, the cardiovascular control conditions and the automatic measurement of BP may be crucial and demands more precise and even invasive devices [58,59]. More sophisticated approaches make use of sensors networks to detect the conditions of hypertensive patients [60] and apply artificial intelligence techniques to achieve more precise values [61]. Nonetheless, a consensus conference on HBPM indicated the characteristics of the most appropriate devices [44]. We assume that it is necessary to rely on the physician’s decision in terms of the best equipment to obtain the patient’s BP.

To develop this work, we chose some similar work to observe prior studies and the techniques they used to achieve treatment adherence. The proposed system relies on Android devices and consumer electronic goods that communicate via Bluetooth. The primary function of the system is the creation of reminders using a computer (gateway) that is sometimes used together with Android devices; these reminders are stored locally and in a cloud infrastructure. The patient is notified at chosen times with audio and visual alerts over handheld devices or TV sets. Other authorized users can also access the information generated via a web application to verify and intervene in the treatment adherence.

## 3. Materials and Methods: Ubiquitous Treatment Adherence System

The objective of this work was to model and build an intelligent system capable of assisting patients with the ingestion of drugs and promoting their adherence to treatment. Additionally, the regular measurement of blood pressure from patients, following the actual medical consensus, was added to the system as a feedback of the patient’s condition.

**Figure 1:**
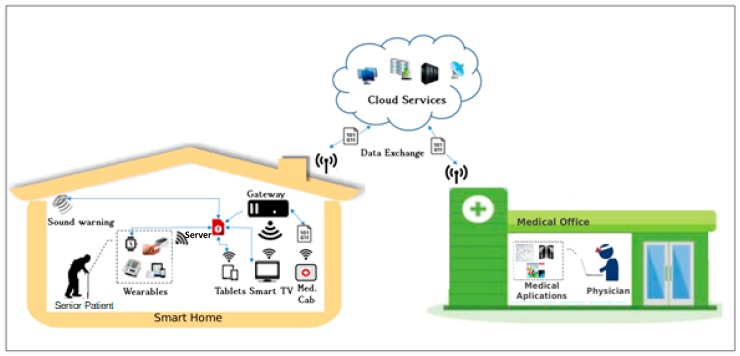
Overview of the system. The scenery is divided into two parts, the smart home and the medical office; they are connected by the cloud infrastructure.

The scenario presented in Figure 1 shows the components of the proposed system. The system is designed to serve an elderly patient living alone or with a partner, in one residence connected to the Internet. Patients are monitored by health teams and relatives passively, meaning that only in exceptional situations does a warning transmit to these supporters. The residence has IoT equipment connected to a server; smartphones; at least one smart TV; other gadgets, such as smart watches; and a blood pressure meter (also connected to the server). Feedback about the right medication is delivered by one smart medicine cabinet and by dialog screens in smartphones or smart TVs.

The smart TV plays a crucial role in this system, as it is a popular system available in many homes. Among the most promising applications provided by interactive digital television systems (iDTV) are the ones that enable TV sets to work as the central processing unit of a house. This unit is also known as a gateway. These extensions are supported by the extending of iDTV’s standard functionalities, making use of its processing power to execute interactive software applications. Our group developed an infrastructure that allows the exchange of data from the iDTV to home automation devices, such as sensors and mobile devices. The integration process among the involved technologies, as well as each element in the proposed infrastructure, is described in [62]. This model is supported by the Open Services Gateway Initiative (OSGi), as a framework for networked devices, and the Brazilian reference middleware, Ginga. Thus, it is possible to use device services in OSGi-based home networks through the digital TV’s interaction with mobile phones and electronic sensor devices using WiFi and Bluetooth technologies [63]. We also developed an API that integrates iDTV applications and home network services. The idea behind the API model is to export service objects from one side to another, allowing the development of two types of applications: the first one on the Ginga-J machine and the second one on the Ginga-NCL machine.

One initial prototype made use of this consumer electronic device to inform the cardiac frequency in real time. This information was presented whenever the system was requested and in particular situations when limit values were reached. In the current version, the TV is used as one of the dialog interfaces for periodic measurements. The system demands from time-to-time, following a predefined schedule, the measurement of the user’s blood pressure and these warnings are made frequently over the smart TV.

With respect to the location of the user, the system needs to basically identify if the user is at home or not. This localization is used to determine which device will primarily be chosen as communication media with the patient. In case the patient is not at home, our system uses mobile communication alternatives. When the patient is at home, an additional localization routine is triggered and will try to determine in which room and to whose device the alert for taking medicine shall be sent.

In other words, the system is smart enough to detect which specific device is being used. For example, if two smart TVs are on, one located in the living room and another one in the bedroom, the system will search for the current location of the patient to define to which TV set the warning should be sent. In case the user is not at home, or if no TV set is on, the notification is sent to the patient’s smartphone.

In the scenario presented, the patients also make use of a system of medication-taking reminders, which, through wireless technology, informs a gateway of the dates, times, and locations of medication intake. After processing at the decision layer, the doctor and relatives are sent notifications via the cloud, allowing them to track adherence.

The local and remote monitoring of the ingestion of prescribed medications is evaluated in real time, and the physician is informed through private SMS messages and social networks if the patients are not following the medical prescription, or if they do not measure the BP accordingly. These procedures allow the doctor to intervene to improve adherence to treatment.

Thus, a scenario was proposed to verify and test an automated residence with a smart medicine cabinet and other Internet-connected devices that promote adherence. The situation for the smart medicine application consists of a software architecture that integrates sensors in the residence to monitor medicine intake according to electronic prescriptions, which are managed locally by a computer connected to the cloud.

All modules are designed to integrate with other technologies and are connected to an IoT infrastructure that allows any device to be connected to the Internet, for which the device must have a valid IP. In our work, we assume that all the equipment of the residence is connected to the IoT gateway.

**Figure 2:**
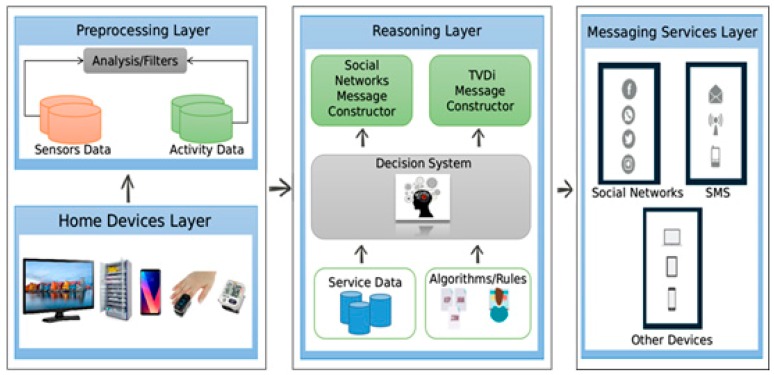
System architecture proposed to process data and send social network messages. There are four layers, which are responsible for data gathering, analysis, decision making, and messaging.

The proposed architecture is divided into four interconnected layers that can receive input data acquired by sensors and various devices for preprocessing and decision making. The medicine cabinet is considered an independent subsystem and is treated as another residence data generator. Figure 2 shows the architecture layers that will be discussed in the following subsections.

### 3.1. Home Devices Layer

The home devices layer consists of several sensors, including cameras, presence sensors, luminosity sensors, and thermometers, as well as devices belonging to the residence, such as computers, tablets, smart TVs, smartphones, smartwatches, one connected blood pressure meter, and one medicine cabinet, which have the tasks of collecting data from the environment and the user.

Examples of data that can be collected in the environment are the inputs and outputs of a specific patient’s movements. The results of a door-opening and door-closing sensor and a micro camera can be captured and stored in the database. Other examples are sensors directly attached to electronic locks, relays, remote controls, and triggers. Information from them is stored in the database in the preprocessing layer and serves as a starting point to identify patterns of access for each room in the house after analyzing and filtering the data. Table 1 presents an example result from a database consisting of an array of events. The data were collected from two door-opening sensors, D1 and D2, which, respectively, represent the door of a room and the medicine cabinet door. The healthcare and activity dataset consist of attributes that can represent people, physical objects, specific locations, medical prescriptions, and warnings sent by the medicine cabinet, by using a smartwatch, smartphone, or smart TV.

Table 2 shows the executed actions, dates/times, locations with longitudes and latitudes, and users’ identifications. The dataset was made into a readable document by the WEKA data mining and machine learning tool [41]. By observing the data extracted in Table 2, we can determine the target attribute. For example, an observation based on the schedules described for each user makes it possible to check the entries in the residence and the opening of the medicine cabinet door. Both items of information together may infer that the medication was taken at a specific time.

With more data and more attributes, it is possible to estimate precisely whether the user takes his or her medicines correctly. Thus, the decision system can consider the contextual data of user actions. In addition to residence sensor data, there are also data collected by the medicine cabinet based on electronic medical prescriptions. Table 3 contains a sample of these attributes.

In the example in Table 3, the data were collected by various systems, including the medicine cabinet, mobile applications, and medical office applications. The schedule for taking medicines starts from the first alarm that stores the return given by the software application and sends it to the decision system. Depending on the situation and on the patient’s location, warnings or alarms can be sent to a TV set, tablet, smartphone, smartwatch, social networks, etc.

### 3.2. Preprocessing Layer

Data characterization for preprocessing consists of mapping the attributes of the dataset objects—their types and whether they are qualitative or quantitative—and defining the operations that can be performed on the attribute values. The scale can be nominal, rational, or interval [64]. The analysis consists of preparing the data to describe the objects using a character vector or a set of input attributes. For example, a GPS object on a smartphone has the characteristics of collecting location coordinates, and the date and time.

This layer is also responsible for demanding the specific measurement as the periodic information about the blood pressure of a patient. Based on the medical prescription, this layer will request the collection of these data. This demand is processed by the home devices layer that will pop-up a message on the smart TV, send an SMS, or send another message on the tablet according to the patient context.

From the analysis and filtering of these data, using machine learning tools and algorithms, we can define the times that a patient usually is in his or her residence, and thus discover his or her pattern of location. The data analysis and filtering presented in the preprocessing layer are done to extract the necessary characteristics to identify groups and similar objects in the dataset, along with association rules that relate these groups. This layer helps with preparing the context and the bases of the devices. The filter used to improve the database was adapted from the work of Chandrasekar and teammates [40]. It consists of transforming nominal attributes into binary attributes to enhance classification in terms of the target attribute using a decision tree.

### 3.3. Reasoning Layer

The reasoning layer represents the intelligence of the system and is divided into two subsystems, which are the rules built by specialists to aid in decision making and algorithms with strategies and pre-established practices that also assist in decision making. The generalization of examples defines learning problems. The system can formulate models as search problems over a space of possible solutions.

**Figure 3:**
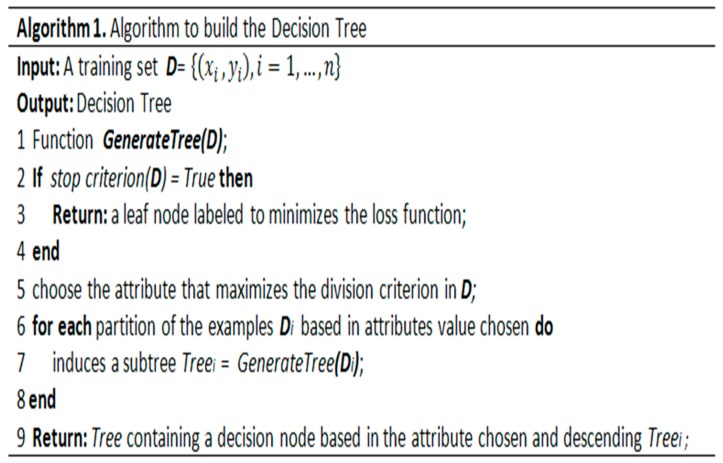
Generic decision tree algorithm.

Our approach implements C4.5, RandomTree, and RepTree [65], which construct models based on binary rules. We use the output of the algorithms to generate automatic services for health teams and relatives to accompany the patient’s treatment. The generic algorithm for constructing a decision tree was adapted from Cramér [66] and presented in Figure 3.

The algorithm receives a training set D in the input GenerateTree function and returns a decision tree in the output. The algorithm evaluates the stopping criterion, and if further divisions of the dataset are required, an attribute that maximizes some measure of impurity is chosen in step 5. In step 7, the GenerateTree function is called recursively for each partition of the dataset D. Finally, in step 9, the algorithm returns the tree containing a decision node based on the chosen attribute.

The algorithm uses entropy to build a decision tree from a set of training data (S = s_1_, s_2_, … s_n_) consisting of classified samples. Each sample dataset consists of a one-dimensional vector that represents the values of the attributes or characteristics of each sample and the category or class to which it belongs.

Entropy measures the information gain and determines which attributes are most likely to be needed in the classifier [65]. Equation (1) shows the overall calculation of entropy, which is based on the sum of the probabilities of the attributes as follows:

(1) $$H\left(A\right)=\;-\sum iPi\times \log_{2}\left(Pi\right)$$

Assuming that the probability of observing each value is *P*_1_, *P*_2_, …, *P*_n_ with domains *A*_1_, *A*_2_, …, *A*_n_, the entropy is calculated to use the best attributes to improve information gain and avoid wasting memory and processing power on the treatment of a great mass of data, in addition to reducing noise in the database.

Decision trees are simple and readable data structures that express their results very clearly. They can be easily understood and applied to massive datasets and can manipulate continuous and discrete attributes. Thus, the decision subsystem, through established policies and strategies, defines which services can be made available to the user based on its current perception of history, which is derived from applying the model generated by the algorithms.

The schedule of the prescribed medicine, as well as for the BP measurement, is treated by this layer, which is responsible for recalculating the plan whenever mistakes are detected (for example, losing scheduled doses). The designed algorithms follow good medical practice and will always contact the physician for unknown procedures. Those algorithms are also responsible for demanding the firing of messages for the users (patients, doctors, caregivers, relatives, etc.).

### 3.4. Messaging Services Layer

The messaging services layer allows the results of the reasoning layer to be used to present processing results to various applications. In the example built for this work, alert applications were developed for social networks, such as Twitter and Facebook, short message applications (SMS), mobile applications, and embedded apps on smart TVs, smartphones, and tablets (over WiFi).

The expected outputs from the system are the services available to the user according to the data analysis. An example is a message that informs the health team that the prescribed treatment is not being followed, leading to the identification of nonadherence and adherence patterns. Another service is the identification of the user’s position to make medication notices and alarms available through household appliances and devices, such as smart TVs, tablets, smart watches, and smartphones in the residence.

Actions determined by processing can be sent to the actuators and communicating devices, thus closing the cycle of acquisition, learning, and execution. Several services can be developed using the architecture presented here, such as systems for access control and residential alarm systems. To that end, one can program policies and strategies based on the analysis of data from the sensors and previously established devices.

## 4. System Implementation

After presenting the conceptual decisions of our system, in this section, we will present the technical choices that allowed us to implement the architecture. We added embedded applications integrated within a smart medicine cabinet, smart TVs, smart watches, and smartphones to check patient coordinates, as well as one certified blood pressure meter connected to the data network. The developed prototypes, hardware, and software are presented in the following subsections.

### 4.1. Smart Medicine Cabinet System

The smart cabinet has an architecture that incorporates radio frequency identification (RFID) tags that are associated with patients. Each patient has a specific prescription that allows the cabinet to generate information, such as alarms and warnings, for other applications to use. This prescription is stored on the home server that shares its contents with the medicine cabinet and with other interface devices, such as smartphones. Modifications will be accepted only when authorized by medical staff.

The cabinet is part of a research and development project in partnership with the University of Stuttgart in Germany. Inside the medicine boxes, there are RFID tags with specific information about each package. The cabinet can check if the carton being removed at each time is the one that contains the prescribed medicine for that time of the day. In case of mistakes, the cabinet emits a high buzz sound and a red-light alarm to warn the patient; when the removed box contains the correct, scheduled medicine, the cabinet is illuminated with a green light (no sound is emitted). These alarms should be enough to prevent the wrong choice of medication. Additionally, the smart cabinet manages two types of medicine, either those that are mandatory or prescription-free medications. Technical information about the implementation of the cabinet may be found in [67].

**Figure 4:**
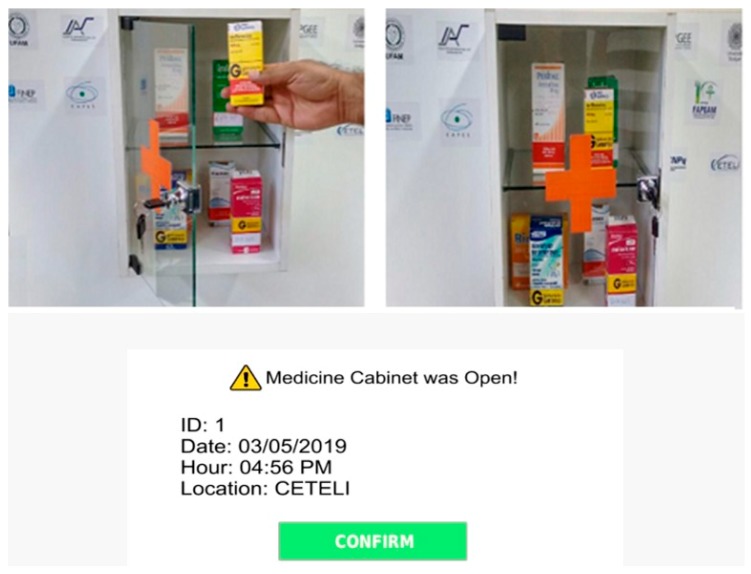
Medicine cabinet system and system log.

In recent years, the cabinet’s functionalities were expanded; now it can connect to the cloud and has a voice synthesizer and a facial recognition system to aid in the collection of patient data. Its application provides real-time logging of the activities identified by the sensors. Figure 4 shows the ID from the patient’s RFID card, the date, time, and location of the cabinet opening, illustrating some of the data collected by the system.

The access control of the medicine cabinet and the entrance door of the research laboratory were developed with two forms of feature redundancy. An aperture sensor and a webcam were also installed at the laboratory to capture input data. In the cabinet, the same approach was taken with aperture sensors, camera identification, and the RFID access card. Thus, it was possible to know if the owner of the card was the same person identified by the camera. The webcam identification system used a set of matching algorithms, a Haar cascade frontal face alt tree [68], and Haar cascade tree eyeglasses [69] that use Fisherfaces methods, which seek a linear combination of the characteristics of the face. We combined the algorithms with a k-nearest neighbor (KNN) algorithm to improve facial identification accuracy [70].

The face recognition algorithm used on the smart cabinet was simplified because the system is aimed at a small number of users. As a system designed for the home, we assumed that no more than eight patients would make use of it, reducing the processing time and effort. The facial recognition technique used was linear discriminant analysis (LDA), also known as Fisherfaces, based on the linear and nonlinear transformations of the coordinate systems [71]. The LDA has as a method to reduce the rays of each class as well as the variance existing in them; this guarantees a better separation between the datasets of each class by applying a linear transformation to find the best coordinate system for the best representation of the data with the maximum separability. From a database, the technique highlights the best vectors that represent the best space model of the whole image that is based on the calculation of eigenvectors and eigenvalues. In practice, each facial image present in the database has the representation of a linear combination of eigenvalues, so the coefficients of the combination will be the new representation of the face. A complete description of the algorithms used, as well as the performance obtained, can be found in the final project report by Santana [72].

### 4.2. Adherence to the Treatment Estimation

Treatment adherence involves many aspects beyond medication ingestion. Not all patients inform their physicians when, for example, a slight side effect occurs. Many of them stop taking the medication until a new symptom forces them to seek a new medical appointment. In some cases, due to either intolerance or allergic effects, it becomes impossible to follow the prescription, and a second or third appointment is needed until the treatment can be rightfully followed.

After consulting and having an agreed prescription to follow, patients must observe the frequency for which the medicine should be taken, the right dosage for each schedule, and the total duration of the medication process. For the relationship between the user and prescription, the right time to take the right medicine is the most relevant aspect. One typical issue that might come up with the medication schedules is what the patient should do when missing the prescribed time to take medicine. The recommendations when such misconduct occurs differ depending on the treatment in four different ways:

1. Patient should ignore the missed schedule and take the medicine at the next schedule as usual, adding the missed schedule to the end of the medication.
2. Patient should take the missed schedule immediately and continue as usual.
3. Patient should take the missed schedule immediately and reschedule the rest, based on the newly taken time.
4. Patient should skip the missed schedule and take a double dosage on the next schedule.

Once again, we affirm that the decision on what to do in each case must be determined by the medical staff. Most of the time, the user does not know all this technical information or neglects it. Our system can deal with these four situations, demanding the doctor’s report of the right procedure.

As seen, there are many parameters to be considered when taking medicines. Concerning the adherence measuring, we adopted the approach presented by Singh and Varshney [73] and calculated their effective medication-adherence proposal. The main inputs to infer the adherence are the number of doses taken, the number of missed doses, and the time variation between doses.

The average medication adherence level during an observed period is given by Equation (2). *N_pres_* is the number of prescribed doses over the given time and is given as *N_taken_*+ *N_missed_*, with *N_taken_* and *N_missed_* representing the number of doses taken and the number of doses missed by the patient, respectively.

(2) $$MA=\;\frac{N_{taken}}{N_{press}}\times 100\;\;$$

Singh and Varshney also affirm that the time variations between doses are also essential values when evaluating the medical effectiveness of doses. They proposed a formula to calculate the probability that the gap between two doses has exceeded the max interdose time given by Equation (3) for k = 0:

(3) $$Prob(\left(T_{I+1}-\;T_{I}\right)>T_{MAX})={\left(\lambda t\right)}^{k}\times e^{-\lambda t}/k!\;\;$$

where $T_{MAX}$ is the maximum time allowed between any two doses to remain medically effective, λ is the ideal rate of the dose consumption event, k is an integer constant (for $T_{MAX}\;\;$, k is equal 0), and t is the time observed. The constant k may also have the value 2, for calculating the minimum interdose time to remain medically safe, and details can be found in [73]. The authors also affirm that adequate medication adherence depends on the average medication adherence and the pattern of adherence. On one hand, taking doses too closely together may not improve their effectiveness due to the increased potential for side effects or overdose, which may reduce medication adherence over time but in some cases, does not affect it. On the other hand, when doses are taken too far apart, the effectiveness of the treatments is definitely reduced. That is why they decided to use the pattern only when doses were taken too far apart for adequate medication adherence, resulting in Equation (4):

(4) $$EMA=MA-Prob\left(\left(T_{I+1}-\;T_{I}\right)>T_{MAX}\right)\times MA\;\;$$

Following this concept, it is possible to know if the expected doses were all ingested or not. However, the time of ingestion and the measured interval between doses is what will determine the degree of adherence to the treatment. Our system can record that information automatically when the patients are at home through the medicine cabinet. When they are away from home, they will be alerted about their medication through the mobile device, and the only alternative we have is to rely on the information recorded by the patients, relying on their commitment to the treatment.

### 4.3. Localization of the Patients

The first localization routine will obtain the global location of the patients and is supported by their smartphone positioning system (GPS based). Considering predefined known coordinates, such as a workplace, supermarket, or club meeting room, we can collect data on the patient’s behavior and location. The most important coordinates known are those of the patient’s own home.

For the in-house scenario, the location routine needs to decide where to send the desired message, and it makes use of the API that integrates iDTV applications and home network services. Each TV set running at that moment is registered as an available communication service. If only one TV set is on, it will be the primary communication device and will serve all inhabitants of the house. In case more the one TV set is running, our system needs to determine each one that our patient is attending. In that case, additional presence and brightness sensors spread on the environment being monitored, together with the media access control address (MAC address) of their registered mobile device, helps to improve the patients’ localization. The triangulation of the WIFI signal (received signal strength indicator - RSSI strength) from each connected MAC address allows reaching the approximate location with an accuracy of 2 to 5 m [74,75,76], which should be enough for our application.

Precisely, for this work, we made use of a hybrid indoor location algorithm, such as the one proposed by the group of Li [77] and implemented by our group in previous research. One radio frequency classifier stores the information of the identifier of the patient (based on the mobile phone), the received signal strength (RSSI), and the signal-to-noise ratio (SNR) to determine the location. At each place, the collected data are submitted to a fusion algorithm with an indication of approximate distance and direction about the network node.

The proposed mapping is based on the use of the signal power values for the identification of the distance between the processed mobile device and the access point. Additionally, it is necessary to address the attenuations caused by numerous factors to obtain the signal attenuation between the mobile device and the access point by Equation (5):

(5) $$A_{ar}=20log\;\frac{4\pi D}{\beta }\;\;$$

where $A_{ar}$ is the attenuation in dB, *D* is the distance in meters, and β is the size of the wave period in meters. With this information, it is possible to isolate the distance value as the variable to be determined from the power of the transmitted signal. To calculate the signal power received, considering the influences of the obstacles for the determination of the distance, we used Equation (6):

(6) $$R_{x}=\;P_{t}+G_{ar}-A_{ar}\;$$

where $R_{x}$ is the power of the signal in dB, *Pt* is the transmission power—measured in dBm, and $G_{ar}\;\;$ —the transmission antenna gain, measured in dBi. The system calculates the direction where the centroid is (where the signal is most potent) by measuring the signal’s length. This calculation is carried out continuously and allows for estimating the position of the device about a fixed node. RSSI is expressed in decibels from 0 (zero) to −120 (one hundred and twenty) dB. The closer it is to zero, the stronger the signal. Signal quality is a percentage value between 0% and 100%, where the numbers closest to 100% have better quality. For RSSI values lower than −80 dB, the noise may make it unfeasible. The SNR is obtained by the power ratio of a signal (meaningful information) and background noise (unwanted signal).

A set of tests was realized, and the location obtained was sufficient for the purpose of this work, considering the limits of a conventional house. The location-precision acquired was better than 2 m. These results, as well as a comparison with other related works, can be found in [77,78]. In fact, this location algorithm has already been applied to other applications developed in our research group, such as a teleoperation system of an electric wheelchair [79].

### 4.4. Warnings and Events Visualization System

One smart TV warning application was developed in the Lua and NCL programming languages to allow portability to the Brazilian digital TV system. This system is available in every TV model in Brazil. This solution allows the patient to view his or her medication-taking notices according to the prescription. The purpose of the application is to collect the date and time the patient took the medication and whether he or she took it late or stopped taking it. The app allows the patient to inform others whether the drug was taken by employing two buttons that can be clicked using the TV’s remote control.

**Figure 5:**
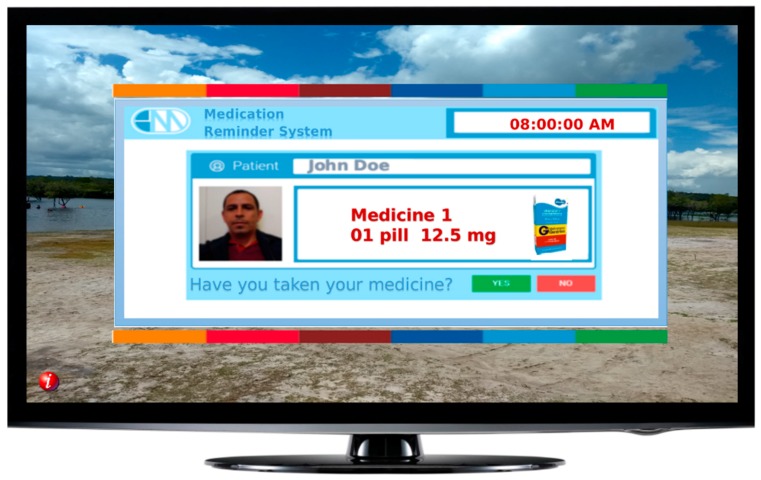
Smart TV warning for medication-intake example.

Delay is computed if the patient takes thirty minutes or longer after the warning is displayed on the screen. This same warning is sent via the network to the gateway store in the database and generates external services, such as messages in XML format. These messages can be SMS alerts or individual posts on Twitter or Facebook. Figure 5 shows a TV alarm example containing prescription data. In this case, an XML document was generated and sent to the gateway for processing to the TV option.

Similarly, a warning message concerning the prescribed schedule is sent to the TV-set asking the user to measure the blood pressure value. In fact, the value is automatically registered as soon as the meter finishes the measuring procedure. These values are used as input data in the reasoning layer that can decide the course of action in cases of missing the measurement and the nonreactive behavior of the patient.

**Figure 6:**
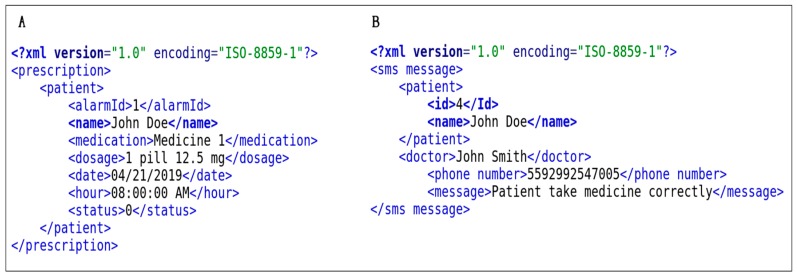
Warning and message data in XML format.

The gateway interprets the XML document generated and decides where to send; it can identify that the patient is watching TV and commands a pop-up message such as the one in Figure 5, or it can trigger an SMS message and send it through the cell phone provider, or even as a direct message to the physician’s social networks. Figure 6 contains an insert of the XML document generated, where Figure 6A shows the data interpreted by the TV as it appears in the application, and Figure 6B presents an example of an SMS message generated by the gateway.

**Figure 7:**
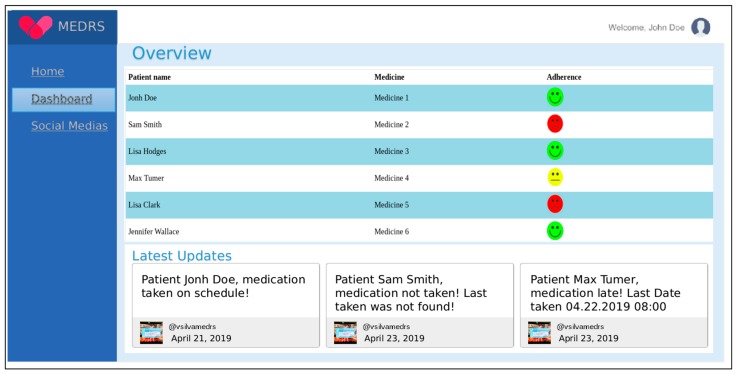
Mobile website integrated with Twitter.

The mobile Web system consists of a patient registration system and a visualization of events occurring in residence. It automatically posts messages to social networks based on the decision system. For example, if the patient does not measure blood pressure as prescribed, messages will be fired to relatives about this issue. In Figure 7, the main website and notification screen on the social network Twitter are presented through a Twitter bot. To this end, an integrated module was developed for social networks to automatically post warnings, which allows relatives and health staff to accompany the patient during treatment. The messages are private; only the registered medical staff and authorized relatives can access them through the access key of the social network API.

### 4.5. Decision-Making System

Adherence measurement is conducted as follows. The first task is to collect the patient’s drug ingestion pattern data and compare it to the prescription data. Divergences are pondered. It is necessary to infer the degree of adherence to create a training basis based on the collected data or use a free basis for research purposes. After applying filters for the removal of duplicate attributes, an algorithm that best fits the problem is chosen; we chose the decision tree algorithm. The system is trained with the database to identify the effectiveness of the algorithm, and then the decision model is implemented that behaves best with the data. As the problem was classified as a binary decision, in terms of adheres or does not adhere, after the implementation of the model, it was expected that the behavior of taking medication would be identified quickly in real time, informing whether the patient is adhering or not to the treatment based on the evaluation of every intake of prescribed medicines.

**Figure 8:**
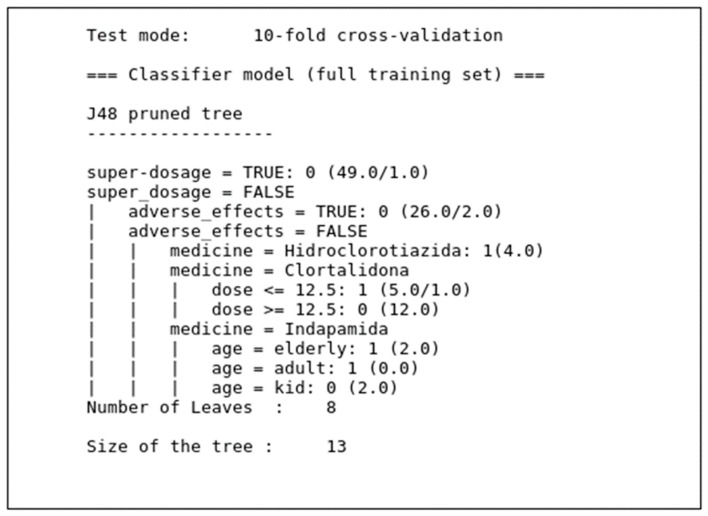
Decision tree from the J48 model.

The decision subsystem was built in two stages. First, the database was verified by three decision tree-based classifiers that extracted the rules. For this stage, we used WEKA software, which already contains several algorithms, including those used in this work. Then, based on the model generated by WEKA, a decision module was built and incorporated into the system to classify data at runtime. Figure 8 presents the decision tree generated by the J48 algorithm for the classification of medication intake for hypertension. The model was based on the choice of the best attributes for classification of medication intake.

RepTree and RandomTree algorithms also generated similar trees, but with smaller size and less classification power than that of the J48 algorithm. In addition to the models built for training based on the J48, RepTree, and RandomTree algorithms, we programmed software to classify data at runtime, which allows the trained algorithms to receive data in real time, organize them, and send them to other applications.

**Figure 9:**
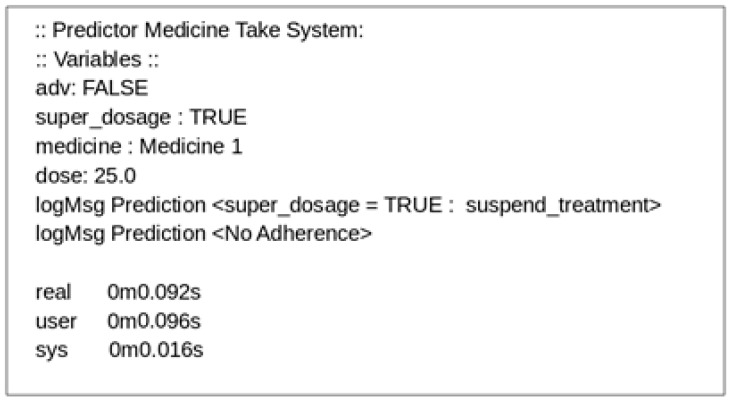
Decision-making system log.

Figure 9 shows the output of the application’s execution, which identifies super dosing and nonadherence by the patient. The execution time of the algorithm was promising, since it was less than 0.1 s, leading us to believe that the response time of this application is sufficient to deliver data to a higher layer of the architecture in real time when considering human behavior.

### 4.6. Blood Pressure Meter

**Figure 10:**
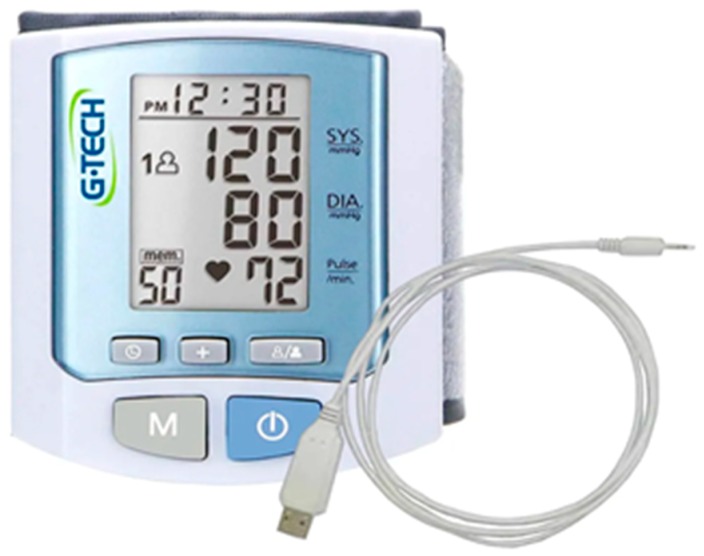
Illustration of the commercial blood pressure meter utilized.

The blood pressure meter chosen uses the oscillometric measurement method with a semiconductor pressure sensor; its measurement range is between 40 and 250 mmHg with pulsation of 40 to 199 beats per minute. It has a USB cable port for transmitting measurement data to a computer and the memory capacity for up to 50 measurements in two user zones. The meter was purchased on the market, and a microcontroller board was built to transmit data from each measurement wirelessly to the residential gateway via a Bluetooth connection. This board is connected to the meter via its USB port. An illustrative picture of the metering device is shown in Figure 10.

## 5. Tests and Evaluation of the Results Obtained

The complete system was intensively tested in the lab of embedded and automated systems at CETELI-UFAM. There, we have access to a server in the cloud and the entire infrastructure of computer networks and distributed sensors. We added two door-opening sensors to the medicine cabinet and another two at the laboratory door. Regarding the two cameras, one was focused at the laboratory entrance door for registering presence, and the other at the medicine cabinet for the facial identification of users. RFID readers were used for checking the prescription cards and detecting medicine box movements from the cabinet. A low-cost computer connected to the cloud was used to synchronize the data collected from the TV, smartphones, tablets, the blood pressure meter, and medicine cabinet.

The tests were set up as follows: First, the prescriptions, medication data, and device data, such as indoor locations, were collected and stored in a dataset by applying instance filters to discretize a range of numeric attributes in the dataset of nominal attributes. After discretization, the J48, RandomTree, and RepTree algorithms were chosen for training using cross-validation with ten folds. This configuration of parameters was done using WEKA software in the preprocessing step.

Our group teammates played the role of patients to test the functionalities of the system and generated part of the data analyzed. We also added the databases presented by Dima and Dediu [80] to validate our system. WEKA native filters were applied to the database to avoid noise; for example, repeating data, missing data, and data with null fields. Next, a file with a “arff” file extension recognized by the WEKA tool was generated, which allows the visualization of the most suitable models and algorithms for the system’s implementation. The use of the aforementioned database allowed us to infer the adherence of medications to serve as parameters for the decision system.

The tests were executed as follows: We ran the J48, RepTree, and RandomTree algorithms in the same preprocessed database containing one hundred collections distributed among elderly patients, adults, and children who were prescribed hypertension medications. After training the base algorithms, we generated the logs of the classifiers and evaluated the result of each algorithm.

The evaluation metric involved observing the size of the tree constructed by each model; the confusion matrix to extract the accuracy, precision, and recall; and the Matthews’s correlation and PHI indices [81,82,83,84]. The data were tabulated in a spreadsheet to generate graphs to observe the results of the algorithms better.

The results obtained during the tests were divided into categories to better follow the behavior of the algorithms used. The three algorithms were tested with and without attribute filters, and the results were different for all of them. We evaluated the effects presented by the algorithms according to Equations (7)–(9), which are, respectively, the accuracy, precision, and recall. We also used the Matthews correlation coefficient presented in Equation (10), which is a quality measure of two binary classifications that can be used even if the classes have quite different sizes, as is the case for our dataset.

The classification frequencies for each class of the model are given, employing the confusion matrix with results that were classified as true positives *tp*s, which occur when in the real set, the class being searched, was predicted correctly; for example, the result that the patient took medicine successfully. The false positive *fp* occurs when the outcome in the actual set of the searched class is incorrectly predicted; for example, the patient did not take the drug correctly, but the model reported that he did take it correctly. The true negative *tn* occurs when, in the actual set, the class we are seeking to predict was predicted correctly; for example, the patient did not take the remedy successfully, and the model predicted that he did not take the medication correctly. The false negative occurs when, in the actual set, the class we are looking to predict was incorrectly predicted. For example, when the remedy was not taken correctly, and the model incorrectly predicted that it was taken. Table 4 summarizes the meanings of *tp*, *tn*, *fp*, and *fn*.

Accuracy is a measure based on the confusion matrix values; *tp* is the number of positive instances, *tn* is the number of false positive instances; and *p* and *n* are the sizes of the sets of positive and negative data, respectively.

(7) $$accuracy=\;\frac{\left(tp+tn\right)}{\left(p+n\right)}\;\;$$

Precision is given by the number of true positive instances divided by the number of true positive instances plus the number of false positive instances, which results in the following:

(8) $$precision=\;\frac{tp}{\left(tp+fp\right)}\;\;$$

The recall is given by the number of true positive instances divided by the sum of the number of true positive instances and the number of false negative instances (Equation (9)). The Matthews correlation coefficient is similar to the *PHI* coefficient given in Equation (10).

(9) $$recall=\;\frac{tp}{\left(tp+fn\right)}\;\;$$

(10) $$\;\;PHI=\frac{\left(tp\;\times \;tn-fp\times fn\right)}{\sqrt{\left(tp\;+\;fp\right)\times \left(tp+fn\right)\times \left(tn+fp\right)\times \left(tn+fn\right)}}\;\;$$

In the final stage of testing, we ran the joint voting algorithms of Kuncheva [84] and Kittler [85], which consist of selecting a set of algorithms to solve a given problem, verifying the error percentage of each one and choosing the one that best classifies the dataset statistically.

The J48 algorithm with nine attributes, cross-validation, and ten folds resulted in a three-node model of a decision tree. This tree classified the data with 99% accuracy. However, we believe that the tree featured overfitting, so we decided to exclude the “situation” and “age” attributes, which were shown to be redundant and unpredictive of classification.

After a new analysis of the results with the filtered data and the same configurations, the J48 algorithm presented a decision tree model with six levels. The percentage of the classified instances was 95.10% after decreasing the overfitting of the model. Using Equations (7)–(10), we observed that the J48 algorithm presented 95.10% accuracy, 92.07% precision, 97.40% recall, and a *PHI* index of 0.90.

The same parameter settings used for the J48 algorithm were applied to RandomTree. We observed that the RandomTree algorithm presented 91.50% accuracy, 87.8% precision, 94.7% recall, and a *PHI* index of 0.83. The experiment was repeated for RepTree, which showed 90.2% accuracy, 87.8% precision, 92.3% recall, and a *PHI* index of 0.81.

The algorithms tested to classify medication adherence performed well in terms of recall, precision, and *PHI* index. The precision metric presented in [82] and [83] is meant to identify how many samples were positively classified. In other words, it is a measure of how accurate the classification is for positive samples, which is precisely the question we wish to answer; this metric is also known as the positive predictive value.

The Matthews correlation coefficient is a quality measure of two binary classifications that can be used even if the classes are of quite different sizes [83]. The index returns a value between −1 and +1, where a coefficient of +1 represents a perfect prediction, 0 a mean random prediction, and −1 an inverse prediction. This statistic is equivalent to the *PHI* coefficient and attempts, as does efficiency, to summarize the quality of the confusion matrix with a single numerical value.

Table 5 shows the results of the evaluations of the three algorithms used in our reasoning module. The values on the table allow us to infer that the best algorithm for the construction of the classifier for the dataset used in this work is J48 because it has the highest accuracy and has the *PHI* index closest to 1, considering the other algorithms tested. Thus, in a voting system in which the algorithms are ranked according to recall and *PHI* precision, the J48 algorithm stands out and will generate the best model to be built in the decision layer of the application.

The accuracy of the system was shown by 95.10% correct answers for the identification of the proper medication intakes, and therefore, adherence to the medication was considered from this percentage; i.e., the patient adheres to the medication from the training set presented, with the accuracy (hits) of 95.10%. The result is binary, and in that case, the response is adherent or not adherent to the drug. By implementing the model presented in the trained decision tree, we obtained a system capable of identifying whether the patient adhered to the medication based on the confidence index of the algorithm without the need for new training.

### 5.1. Comparison with Similar Works

To position our work in relation to other ones, we chose six similar papers and compared the most relevant characteristics of each described work. This comparison is summarized in Table 6, with the last column containing the attributes of our system. For space reasons, each column contains only the name of the first author together with the publication year and the reference number. The items analyzed, and the meanings of the abbreviations used in the table are as follows:

1. Main Characteristics of the System: Here, we searched for the data communication protocols used that could be WiFi (WiFi), Bluetooth (BT), a commercial mobile protocol, such as 3G, 4G, or similar (MP), or a mix of some of them (Mix). Another aspect studied was the type of devices utilized, which could be dedicated (D), commercial ones (C), or both together (BT). The range of usage verified if the system covered only a limited space, such as a house (HL), or if it reached outdoor places, such as a working place and others (OD). Another investigated aspect was if the system was designed to deal with a specific disease (SD) or general ones (GD). The last characteristic was related to the number of users: if the systems were designed for a small group of users (SG), up to 10 users, or a large group (LG)—more than 10 users.
2. Installation Needs: It is possible to learn from the related work how deep the proposed systems depend on uniquely designed installations. We introduced a scale with high (HD), medium (MD), and small/no (ND) dependency to classify each of them. Similar scales were proposed to estimate the expected installation-adaptation costs; they may be based on high (HA), medium (MA), or small/no (NA) adaptation needs; or high (HC), medium (MC), or small/no (SC) costs, respectively.
3. Relation to Patients: Based on the authors’ descriptions of their systems, we inferred how easy each one was for the user to work. The scale proposed was easy (E), somewhat easy (M), or difficult (D). The grade of invasiveness was classified as not invasive (NI), medium invasive (MI), and highly invasive (HI).
4. Accuracy: This topic covers the ability of the system in detecting events, generating events, and determining adherence. Here, the scales for the three issues are automatic (A), semi-automatic (SA), and manual (M).
5. Feedback to Users: Here, we took into consideration to whom the recorded data will be available by assigning their own patients and their relatives and caregivers with yes (Y) or no (N), based on the information described by each group of authors.
6. Smartness of the System: This item deals with the ability of the system to capture events related to the medication process. We looked for the reported ability to detect medicine intake, identify the right medicine to take, and monitor adherence to treatment; each one is assigned with yes (Y) or no (N). The measuring of vital signals was appointed as automatic (A), semi-automatic (SA), or manual (M). Systems that detect and register patients’ data in an auto-sufficient way are considered automatic. When, after alerting the patients to take some action, such as measuring their blood pressure, the data are recorded directly by the system, it was deemed to be semi-automatic. Finally, systems that depend on the patients for both taking the measures and inserting the measured data are classified as manual. In the case of a real-time adaptation of a scheduled prescription, the systems were classified as fixed schedule (FS) when they were not able to vary from a given schedule, or adaptable schedule (AS) when the system could reschedule the prescription after the patient had forgotten to take any medication at the right time. The last characteristic analyzed was whether the proposed system was designed to focus on a particular disease treatment (F) or was concerned with general treatment (G).

A careful analysis of the related papers was done in the sequence and led us to conclude that there are many similar works in use or being developed all over the world. Nevertheless, as a multifactorial phenomenon, medication and treatment adherence are still an interesting research topic, with far from a consensus on how to deal with the relationships patients have to medications and medical staff in order to improve medications’ effects and patients’ benefits.

Leijdekkers and his group from the University of Technology Sydney constructed a prototype for remote healthcare monitoring [37]. Their system’s components communicate over WiFi and Bluetooth and are based on commercial smartphones, wireless sensors, web servers, and IP webcams installed in private homes. Medical staff may access patients’ status from one healthcare computer server extending the usage range. The prototype was designed without focusing on specific diseases and for a large group of users. No dedicated device is needed, but the prototype demands some installation adaption that leads to increased costs. The main component is a smartphone that requires some literacy to handle. The use of indoor cameras makes the system somehow invasive. Events, like patients’ movements or falls, are detected automatically, but some feedback demands the user’s interaction in a semiautomatic behavior. The system communicates with the users and their relatives, but there is no approach to detect the medicine intake nor to identify if the proper medication is being taken following a schedule. Those actions rely on the users, while the measurement of blood pressure is done in a semi-automatic way. There is no mention of any procedure to evaluate if or at which level a patient is following the treatment protocols, in general or for a specific disease, and the system also needs intervention to reschedule prescription times.

A group of scientists headed by De Bleser tested the user performance, satisfaction, and acceptability of a system named Helping Hand™ (B&O Medicom), which was designed to monitor medication adherence [27]. This dedicated system communicates over Bluetooth, being intended to cover a limited space dealing with no specific disease and with a small number of patients. A schedule-programmable blister cardholder device reminds the patient about medicine intake by firing sound signals. Light signals of different colors indicate the level of medication adherence. The system depends entirely on the dispenser, which increases the final costs and installation needs. The dispenser is not invasive at all, and the proper medication intake is registered automatically, but the patients need to learn how to use the dispenser, which must be loaded manually. Feedback is given exclusively to patients by using different lights and sounds; no feedback data was reported. No vital signals are measured, and the system was planned to deal with a fixed schedule. According to the authors, testing the system showed positive usability aspects, but several areas still require technical improvement. Among their concerns are the usability and acceptability based on the weak sound signal, problems loading the medication, and the fact that the device was designed to deal with only one prescribed medicine at a time.

Tang and colleagues from the Northwestern Polytechnical University in Shaanxi described a prototype named multimedia healthcare system (MHS) [13]. The MHS architecture was designed to cover a limited area and a small number of users. The system is also supposed to deal with general diseases. It makes use of various commercial devices communicating among them by WiFi and Bluetooth, and additional sensors are spread on the living ambient to deliver data to the system. It includes an E-Cabinet able to dispense two different medicine pills sensed by RFID tags. This dedicated device demands some installation adaption and increases the installation costs. The system is not invasive and was tested by a small group of subjects that concluded that it was easy to use but presented some issues. For example, their opinions about the automatic detection of medication intake and related events were not accurate. A self-defined algorithm determines the adherence considering frequency and proper time of medicine intake. Patients are informed about the appropriate sequence of treatment, but there is no mention of contact with the patient’s relatives. The system can detect the medication being taken but needs human intervention to change the schedule. Vital signals like blood pressure are not considered, and the system is concerned with general disease treatment.

A group from Proteus Digital Health, Inc., led by DiCarlo, proposed a digital health technology able to determine precisely when medications have been ingested [34]. This information is provided wirelessly in a confidential way to patients and designated caregivers, health providers, and researchers. The system works with diverse communication protocols and is based on an integrated circuit microsensor that must be ingested with the medication, and covers a limited area due to the low signal strength. It was tested with a small group of users with no specific disease. The necessity of one microsensor for each medicine, as well as a dedicated device to detect its intake, increases the costs and the installation adaption needs. Although easy to use, it is highly invasive, as a strange material must be taken together with the medicine. Sensors interact automatically with the system that can evaluate medication adherence without human interference. Patients and relatives, as well as registered healthcare professionals, may receive real-time feedback about the treatment adherence along with physiological parameters to learn body responses to the drug. The medication schedule is fixed, and the system was not designed for considering any measurement of vital signals. Additionally, it is supposed to be used in the treatment of any disease.

Varshney describes a smart medication management system (SMMS), developed at Georgia State University [12], that remotely monitors and regulates patients’ prescriptions. The system makes use of diverse communication protocols, both wired networks and wireless local area and wide area networks. It was designed to support communication with patients and caregivers over commercial devices to improve schedule-keeping. Additionally, an adherence feedback module is available. No specific disease is covered, and it was designed to work with a small group of users. The prototypes described use sensors to obtain patients’ information, such as vital signals and other biomedical parameters; nevertheless, the usage of specific devices is limited to the medicine dispenser. The system’s functionality depends on several sensors spread across a smart home’s infrastructure, which increases the installation demands and costs. The complete system predicts the use of wearables, increasing its invasiveness. A self-defined model is applied to determine the adherence level of each patient automatically. No mechanism guarantees that the medication is taken accordingly, and the motivation and even the rescheduling of medicines depend on the caregiver personnel. No vital signal measurements are predicted.

Tschanz led a group of researchers that developed an electronic medication management assistant (eMMA) at the Bern University of Applied Sciences [29]. The objective of their proposal is coaching patients, living in private homes, improving their medication intake precision, and reducing misuse. The solution is based on a smartphone application, and its functionality relies on Internet availability; i.e., on the use of commercial devices and diverse communication protocols. No special devices are used, thus minimizing the expected costs and installation needs. The effectiveness of this non-invasive system depends on the user’s ability to communicate by written messages via mobile phones. The detection of relevant events depends on the patients’ responses, but the generation of alerts by the system is done automatically. These alerts may be sent to caregivers. No medicine dispenser is used, limiting proper medication identification and precise intake time. Adherence is measured based on the patient’s feedback in a semi-automatic way. Additionally, the proposed algorithms avoid contraindications and medication errors. The rescheduling of medication is done without human intervention, but the attending doctors must agree to share information with the app. No specific disease treatment is mentioned, and the system does not make use of vital signals.

We conceived and implemented a system based on commercial devices able to improve the treatment adherence of a group of users; namely, the hypertensive patients. The key results obtained may be summarized as follows. As described in Section 3 and Section 4, our system is based on diverse communication protocols, depending on the native connection implementations of the commercial devices used. The proposal was designed to work with a small group of users, considering a typical home in medium and large cities all over the world. In fact, the patients may be reached, sending their data or receiving feedback, inside and outside their homes. Inside homes, the communication is made by conventional home network connection protocols and outside the home by mobile phone ones. One dedicated device is needed: one smart medicine cabinet specially designed for this system. The system is constructed based on components, which makes the medicine cabinet easily substituted by other medicine dispensers with little adaption work. This device is the more expensive component. The other commercial components demand medium installation adaption and have a low cost. We estimated an average price for all components together. Our systems make use of digital TV and smartphone applications to communicate with users, which are very popular and easy-to-use communication devices nowadays. Besides, those mechanisms are also used to give feedback to patients and their relatives, caregivers, and medical staff about their treatment adherence.

The system can detect events to build pertinent context analyses, including presence in a specific room, detection of running equipment, or determination of the most appropriated communication mechanism for one particular situation. The medicine identification and its proper intake are detected by the smart cabinet. Any misconduct regarding the intake schedule leads to a new timetable calculated in real-time and updated immediately on the system. These adaptions consider previous physicians’ recommendations. The system focuses on hypertensive patients and needs to record the patients’ conditions from time to time, so was made respecting the best-known medical practices. At the prescribed moment, the communication devices are activated, staying in this mode until a new blood pressure measurement is made. Adherence is calculated automatically based on a previously-published algorithm considering not only the medication intake but also the registered blood pressure values and the measurement frequency. Unusual or unexpected persistent results are immediately communicated to the medical staff that may interact with the patients and their families for a prompt intervention concerning the treatment. That set of characteristics of our system makes it unique when compared to the related work.

## 6. Conclusions and Future Work

This paper described a system designed to increase the treatment adherence for hypertension patients. Different from existing systems, our proposal is constructed by using commercial consumer electronic devices, and may be easily replicated in any home utilizing a standard personal computer and Internet access. However, the medicine cabinet described, which is commercially unavailable, is the only exception.

The system’s architecture, with four layers, was built to allow the incorporation of devices into an application layer for electronic messages. Based on the medical prescription, the system fires diverse communication alerts to warn patients about the correct intake of medication. The primary interface device is a TV set that advises, through well-designed messages, what the patients should do at that time; e.g., take medicine or measure blood pressure. A collection of alternative communication approaches was incorporated into the system that can also reach the patient through a message to one smartphone or reach relatives and caregivers through social networks.

The contact with medical staff and relatives concerning the patient’s treatment adherence is controlled by a reasoning layer that decides the course of action based on the data collected. It is implemented as a decision tree-based system that classifies medication adherence by using a medicine intake database based on a supervised method. It also considers the periodic measurement of blood pressure and observes the values obtained before deciding how to act. The system was developed based on the J48, RepTree, and RandomTree algorithms to model the decision layer and automatically classify whether the patient adhered to the medication.

The innovation of the work is in the integration of commercial equipment in residences to accompany patients under medical treatment. This connection can be extended to a medical clinic or a cloud data center. Artificial intelligence techniques were used to classify real-time medications taken and the measurements of vital signs of patients being treated to monitor their adherence to treatment. Relatives and health teams can follow up through mobile devices, social networks, and messaging systems in an automated way. The system has great potential to be applied in homes with unaccompanied elderly patients and can be used as a tool for decision making in treatment intervention. Although other schemes described in the literature have similar functionalities, there is no way to compare the results, since those works are based on proprietary solutions and are challenging to reproduce. In contrast, our work is based on low-cost commercial devices.

For future work, we intend to test other approaches using unsupervised methods to determine if they can improve classifications. We also plan to enlarge the database and operationalize it in hospitals and health centers. A new version of the medicine cabinet is under construction and will contain one personalized advice system based on a digital Avatar that will interact with the patient. The concept is to perceive the actual user’s mood, and based on psychological techniques, increase the medication intake’s correctness, as well as the precise measurements of physiological parameters related to the treatment.

The system is not ready for clinical application currently. Nevertheless, the actual use of this system in a set of homes is in the plan. We believe that it is entirely appropriate to be used by a single household or by a collection of homes, similar to the family doctor approach with a small group of health care professionals supervising a part of the local population. After this step, we also plan to study and increase the scalability of our system. Further, it is our unremitting belief that the resulting system should make use of commercial devices.

## Figures and Tables

**Table 1:** Home health monitoring solutions.^1.^

Proposed Approaches	Overview of Main Characteristics	Literature
Smart Homes	Smart home infrastructure with strategically positioned sensors for patient monitoring and improvement of medication adherence.	[12]
	Based on television communication and an electronic medicine cabinet, MHS provides adaptive services for patient monitoring.	[13]
	Personalized home care system integrating wireless sensors, smartphones, webservers, and IP webcams for patient telemonitoring.	[37]
Mobile devices and applications	A health telemonitoring system prototype incorporates an Android smartphone, acting as a gateway between a set of wireless medical sensors and a data server.	[10]
	Data security in Android-based devices used for telemedicine approaches is addressed.	[11]
	A schedule programmable blister card holder device reminds the patient about medicine intake with sound signals. A light signal of different colors indicates the level of medication adherence.	[27]
	Mobile application with a conversational interface improving patient education and informing both patient and health caretakers about medication schedules, intake, side effects, and food interactions, among others.	[29]
Wearable devices	A smartwatch with embedded movement sensors detects patient behavior as indicators for medication intake.	[30]
	Machine learning algorithms are used to detect natural movements provided by a wearable wristband sensor as indicators of the medication intake activities.	[38]
	A shirt with an array of embedded sensors connected to a central processing unit continuously monitors physiological data of the patient.	[51]
Ingestible sensors	An integrated circuit microsensor ingested with the medication gives real-time information about the treatment adherence along with physiologic parameters to learn the body response to the drug.	[33,34]
	Description of an in vivo communication system between a microsensor embedded in the medication and a patch receiver on the patient skin. Data are available to the involved persons via mobile and Web interfaces.	[31,32]
Implantable sensor	A membrane-type sensor is described as continuous blood pressure monitoring.	[55]

^1^ Health monitoring solutions are organized from less invasive to more invasive.

**Table 2:** Dataset sample from closing and opening the doors.

Action	Date	Time	Latitude	Longitude	ID
D1 open	04/21/2019	07:42:32	−3.0878703	−59.9638596	0.90
D1 close	04/21/2019	07:42:00	−3.0878703	−59.9638596	0.83
D2 open	04/21/2019	08:11:06	−3.0878703	−59.9638596	0.83
D2 close	04/21/2019	08:13:16	−3.0878703	−59.9638596	0.83
D1 open	04/21/2019	07:50:00	−3.0878703	−59.9638596	0.83
D1 close	04/21/2019	07:50:35	−3.0878703	−59.9638596	0.83
D2 open	04/21/2019	16:00:00	−3.0878703	−59.9638596	0.83
D2 close	04/21/2019	16:02:00	−3.0878703	−59.9638596	0.81

**Table 3:** Sample from electronic prescription database.

Medicine	Dosage in Milligrams	Scheduled Time	Ingestion Time	Apps Return
Medicine 1	12.5	08:00:00	08:00:00	True
Medicine 2	25	09:00:00	09:00:00	True
Medicine 3	25	08:00:00	08:00:00	True
Medicine 3	50	08:00:00	null	False
Medicine 4	1.5	10:00:00	10:00:00	True
Medicine 4	1.5	10:00:00	11:00:00	True
Medicine 4	1.5	10:00:00	null	False

**Table 4:** Interpretation of *tp*, *tn*, *fp*, and *fn.*

Predicted Value Real Value	Took Medicine Correctly	Did Not Take Medicine Correctly
**Took medicine correctly**	*tp*	*fp*
**Did not take medicine correctly**	*fn*	*tn*

**Table 5:** Results for algorithms tested.

Algorithm	Accuracy	Precision	Recall	PHI
J48	95.10	92.07	97.40	0.90
RandomTree	91.50	87.08	94.70	0.83
RepTree	98.20	87.08	92.30	0.81

**Table 6:** Comparison of the presented work with similar works.

Comparison Group	Subdivided In	Compared Related Work						
		Leijdekkers 2007 [37]	De Bleser 2010 [27]	Tang 2011 [13]	DiCarlo 2012 [34]	Varshney 2013 [12]	Tschanz 2018 [29]	This Work
Characteristics of the System	Protocols	Mix	BT	Mix	Mix	Mix	Mix	Mix
	Types of Devices	C	D	C	D	C	C	C
	Range of Usage	OD	HL	HL	HL	HL	OD	OD
	Specific Disease	GD	GD	GD	GD	GD	GD	SD
	Number of Patients	LG	SG	SG	SG	SG	SG	SG
Installation Needs	Special Devices	ND	HD	MD	HD	MD	ND	MD
	Installation Adaption	MA	MA	MA	HD	HD	ND	MA
	Expected Costs	HC	HC	MC	HC	HC	SC	MC
Relation to Patients	Ease of Use	M	M	E	E	E	E	E
	Invasiveness	MI	NI	NI	HI	MI	NI	NI
Accuracy	Detecting Events	A	A	SA	A	A	SA	A
	Generating Events	SA	M	A	A	A	A	A
	Determining Adherence	na	na	A	A	A	SA	A
Feedback to Users	Own Patients	Y	N	Y	Y	Y	Y	Y
	Relatives and Caregivers	Y	N	N	Y	Y	Y	Y
Smartness of the System	Detect Medicine Intake	N	N	Y	Y	N	N	Y
	Medicine Identification	N	N	Y	Y	Y	N	Y
	Real-time Scheduling	na	FS	FS	FS	FS	AS	AS
	Measuring Vital Signals	SA	na	na	na	na	na	SA
	Adherence to Treatment	N	N	Y	Y	Y	Y	Y
	Treatment of Specific Disease	na	na	G	G	G	G	F

na: information not available. The abbreviations are detailed in Section 5.1.

## References

[1] Abowd D., Dey A.K., Orr R., Brotherton J. Context-awareness in wearable and ubiquitous computing. Digest of Papers. Proceedings of the First International Symposium on Wearable Computers.

[2] Yaqoob I., Ahmed E., Hashem I.A.T., Ahmed A.I.A., Gani A., Imran M., Guizani M. (2017). Internet of Things Architecture: Recent Advances, Taxonomy, Requirements, and Open Challenges. IEEE Wirel. Commun..

[3] Candan K.S., Li W.S., Phan T., Zhou M., Agrawal D., Candan K.S., Li W.S. (2011). At the Frontiers of Information and Software as Services. New Frontiers in Information and Software as Services, Lecture Notes in Business Information Processing.

[4] Knowles B., Hanson V.L. (2018). The Wisdom of Older Technology (Non)Users. Commun. ACM.

[5] Pal D., Funilkul S., Charoenkitkarn N., Kanthamanon P. (2018). Internet-of-Things and Smart Homes for Elderly Healthcare: An End User Perspective. IEEE Access.

[6] Rosales A., Fernández-Ardèvol M. (2016). Beyond WhatsApp: Older people and smartphones. Rom. J. Commun. Public Relat..

[7] Silva V., Souza V.S., Cruz R.G., Jazdi N., Lucena V.F. MobiHealth: A System to Improve Medication Adherence in Hypertensive Patients. Proceedings of the 8th International Conference on Current and Future Trends of Information and Communication Technologies in Healthcare (ICTH 2018).

[8] Dias D., Cunha J.P.S. (2018). Wearable Health Devices—Vital Sign Monitoring, Systems and Technologies. Sensors.

[9] Bergmann J.H.M., Chandaria V., McGregor A. (2012). Wearable and Implantable Sensors: The Patient’s Perspective. Sensors.

[10] Morón M.J., Luque R., Casilari E. (2014). On the Capability of Smartphones to Perform as Communication Gateways in Medical Wireless Personal Area Networks. Sensors.

[11] Gu J., Huang R., Jiang L., Qiao G., Du X., Guizani M. (2019). Fog Computing Solution for Context-Based Privacy Leakage Detection for Android Healthcare Devices. Sensors.

[12] Varshney U. (2013). A Smart Approach to Medication Management. Computer.

[13] Tang L., Zhou X., Yu Z., Liang Y., Zhang D., Ni H. (2011). MHS: A Multimedia System for Improving Medication Adherence in Elderly Care. IEEE Syst. J..

[14] Majumder S., Aghayi E., Noferesti M., Memarzadeh-Tehran H., Mondal T., Pang Z., Deen M.J. (2017). Smart Homes for Elderly Healthcare—Recent Advances and Research Challenges. Sensors.

[15] Karanasiou G.S., Tripoliti E.E., Papadopoulos T.G., Kalatzis F.G., Goletsis Y., Naka K.K., Bechlioulis A., Errachid A., Fotiadis D.I. (2016). Predicting adherence of patients with HF through machine learning techniques. Healthc. Technol. Lett..

[16] Ferdinand K.C., Senatore F.F., Clayton-Jeter H., Cryer D.R., Lewin J.C., Nasser S.A., Fiuzat M., Califf R.M. (2017). Improving Medication Adherence in Cardiometabolic Disease: Practical and Regulatory Implications. J. Am. Coll. Cardiol..

[17] Alfian G., Syafrudin M., Ijaz M.F., Syaekhoni M.A., Fitriyani N.L., Rhee J. (2018). A Personalized Healthcare Monitoring System for Diabetic Patients by Utilizing BLE-Based Sensors and Real-Time Data Processing. Sensors.

[18] Holmes M.S., D’arcy S., Costello R.W., Reilly R.B. (2014). Acoustic Analysis of Inhaler Sounds from Community-Dwelling Asthmatic Patients for Automatic Assessment of Adherence. IEEE J. Transl. Eng. Health Med..

[19] Nousias S., Lalos A.S., Arvanitis G., Moustakas K., Tsirelis T., Kikidis D., Votis K., Tzovaras D. (2018). An mHealth System for Monitoring Medication Adherence in Obstructive Respiratory Diseases Using Content Based Audio Classification. IEEE Access.

[20] World Health Organisation (2003). Adherence to Long Term Therapies: Evidence for Action. Chapter 1, pp. 3–5. https://www.who.int/chp/knowledge/publications/adherence_full_report.pdf?ua=1.

[21] Gimpel G., Varshney U., Ahluwalia P. (2016). Emerging IT for Medication Adherence. IT Prof..

[22] Jimmy B., Jose J. (2011). Patient Medication Adherence: Measures in Daily Practice. Oman Med. J..

[23] Mrosek R., Dehling T., Sunyaev A. (2015). Taxonomy of health IT and medication adherence. Health Policy Technol..

[24] DiBonaventura M., Gabriel S., Dupclay L., Gupta S., Kim E. (2012). A patient perspective of the impact of medication side effects on adherence: Results of a cross-sectional nationwide survey of patients with schizophrenia. BMC Psychiatry.

[25] DiMatteo M.R. (2004). Social Support and Patient Adherence to Medical Treatment: A Meta-Analysis. Health Psychol..

[26] Alexa I.D., Prada G.I., Donca V.I., Mos L.M., Alexa O. Improving quality of life of elderly people aged 85 and older by improving treatment adherence. Proceedings of the E-Health and Bioengineering Conference (EHB).

[27] De Bleser L., Vincke B., Dobbels F., Happ M.B., Maes B., Vanhaecke J., De Geest S. (2010). A New Electronic Monitoring Device to Measure Medication Adherence: Usability of the Helping Hand ™. Sensors.

[28] De Bleser L., De Geest S., Vandenbroeck S., Vanhaecke J., Dobbels F. (2010). How Accurate Are Electronic Monitoring Devices? A Laboratory Study Testing Two Devices to Measure Medication Adherence. Sensors.

[29] Tschanz M., Dorner T.L., Holm J., Denecke K. (2018). Using eMMA to Manage Medication. Computer.

[30] Kalantarian H., Alshurafa N., Sarrafzadeh M. (2016). Detection of Gestures Associated with Medication Adherence Using Smartwatch-Based Inertial Sensors. IEEE Sens. J..

[31] Dua A., Weeks W.A., Berstein A., Azevedo R.G., Li R., Ward A. An In-Vivo Communication System for Monitoring Medication Adherence. Proceedings of the 2017 IEEE Wireless Communications and Networking Conference (WCNC).

[32] Weeks W.A., Dua A., Hutchison J., Joshi R., Li R., Szejer J., Azevedo R.G. A Low-Power, Low-Cost Ingestible and Wearable Sensing Platform to Measure Medication Adherence and Physiological Signals. Proceedings of the 40th Annual International Conference of the IEEE Engineering in Medicine and Biology Society (EMBC).

[33] Hafezi H., Robertson T.L., Moon G.D., Au-Yeung K., Zdeblick M.J., Savage G.M. (2015). An Ingestible Sensor for Measuring Medication Adherence. IEEE Trans. Biomed. Eng..

[34] DiCarlo L., Moon G., Intondi A., Duck R., Frank J., Hafazi H., Behzadi Y., Robertson T., Costello B., Savage G. (2012). A Digital Health Solution for Using and Managing Medications: Wirelessly Observed Therapy. IEEE Pulse.

[35] Hasan M.K., Shahjalal M., Chowdhury N.Z., Jang Y.M. (2019). Real-Time Healthcare Data Transmission for Remote Patient Monitoring in Patch-Based Hybrid OCC/BLE Networks. Sensors.

[36] Baig N.M., Gholamhosseini H. (2013). Smart Health Monitoring Systems: An Overview of Design and Modeling. J. Med. Syst..

[37] Leijdekkers P., Gay V., Lawrence E. Smart Homecare System for Health Tele-monitoring. Proceedings of the First International Conference on the Digital Society (ICDS’07).

[38] Hezarjaribi N., Fallahzadeh R., Ghasemzadeh H. A machine learning approach for medication adherence monitoring using body-worn sensors. Proceedings of the Design, Automation & Test in Europe Conference & Exhibition (DATE).

[39] Phan N., Chun S.A., Bhole M., Geller J. Enabling Real-Time Drug Abuse Detection in Tweets. Proceedings of the IEEE 33rd International Conference on Data Engineering (ICDE).

[40] Chandrasekar P., Qian K., Shahriar H., Bhattacharya P. Improving the Prediction Accuracy of Decision Tree Mining with Data Preprocessing. Proceedings of the IEEE 41st Annual Computer Software and Applications Conference (COMPSAC).

[41] Hall M., Frank E., Holmes G., Pfahringer B., Reutemann P., Witten I.H. (2009). The WEKA data mining software: An update. SIGKDD Explor. Newsl..

[42] Myers M.G., Godwin M., Dawes M., Kiss A., Tobe S.W., Kaczorowski J. (2010). Measurement of blood pressure in the office: Recognizing the problem and proposing the solution. Hypertension.

[43] Filipovsky J., Seidlerova J., Kratochvil Z., Karnosova P., Hronova M., Mayer O. (2016). Automated compared to manual office blood pressure and to home blood pressure in hypertensive patients. Blood Press.

[44] Parati G., Stergiou G.S., Asmar R., Bilo G., De Leeuw P., Imai Y., Kario K., Lurbe E., Manolis A., Mengden T. (2008). European Society of Hypertension guidelines for blood pressure monitoring at home: A summary report of the Second International Consensus Conference on Home Blood Pressure Monitoring. J. Hypertens..

[45] McManus R.J., Mant J., Bray E.P., Holder R., Jones M.I., Greenfield S., Kaambwa B., Banting M., Bryan S., Little P. (2010). Telemonitoring and selfmanagement in the control of hypertension (TASMINH2): A randomised controlled trial. Lancet.

[46] McManus R.J., Mant J., Haque M.S., Bray E.P., Bryan S., Greenfield S.M., Jones M.I., Jowett S., Little P., Penaloza C. (2014). Effect of self-monitoring and medication self-titration on systolic blood pressure in hypertensive patients at high risk of cardiovascular disease: The TASMIN-SR randomized clinical trial. JAMA.

[47] Bliziotis I.A., Destounis A., Stergiou G.S. (2012). Home versus ambulatory and office blood pressure in predicting target organ damage in hypertension: A systematic review and meta-analysis. J. Hypertens..

[48] Ward A.M., Takahashi O., Stevens R., Heneghan C. (2012). Home measurement of blood pressure and cardiovascular disease: Systematic review and meta-analysis of prospective studies. J. Hypertens..

[49] Arakawa T. (2018). Recent Research and Developing Trends of Wearable Sensors for Detecting Blood Pressure. Sensors.

[50] Williams B.R.Y.A.N., Mancia G., Spiering W., Agabiti Rosei E., Azizi M., Burnier M., Clement D.L., Coca A., De Simone G., Dominiczak A. (2019). ESC/ESH Guidelines for the management of arterial hypertension. Eur. Heart J..

[51] Pandian P.S., Mohanavelu K., Safeer K.P., Kotresh T.M., Shakunthala D.T., Gopal P., Padaki V.C. (2008). Smart Vest: Wearable multi-parameter remote physiological monitoring system. Med. Eng. Phys..

[52] Ding X.R., Zhao N., Yang G.Z., Pettigrew R.I., Lo B., Miao F., Li Y., Liu J., Zhang Y.T. (2016). Continuous Blood Pressure Measurement from Invasive to Unobtrusive: Celebration of 200th Birth Anniversary of Carl Ludwig. IEEE J. Biomed. Health Inform..

[53] Vashist S.K., Schneider E.M., Luong J.H.T. (2014). Commercial Smartphone-Based Devices and Smart Applications for Personalized Healthcare Monitoring and Management. Diagnostics.

[54] Sato H., Koshimizu H., Yamashita S., Ogura T. Blood pressure monitor with a position sensor for wrist placement to eliminate hydrostatic pressure effect on blood pressure measurement. Proceedings of the 35th Annual International Conference of the IEEE Engineering in Medicine and Biology Society (EMBC).

[55] Kim S., Park J., So S., Ahn S., Choi J., Koo C., Joung Y.H. (2019). Characteristics of an Implantable Blood Pressure Sensor Packaged by Ultrafast Laser Microwelding. Sensors.

[56] Tafreshi A.S., Klamroth-Marganska V., Nussbaumer S., Riener R. (2015). Real-Time Closed-Loop Control of Human Heart Rate and Blood Pressure. IEEE Trans. Biomed. Eng..

[57] Yoo S.Y., Ahn J.E., Cserey G., Lee H.Y., Seo J.M. (2019). Reliability and Validity of Non-invasive Blood Pressure Measurement System Using Three-Axis Tactile Force Sensor. Sensors.

[58] Crimi A., Makhinya M., Baumann U., Thalhammer C., Szekely G., Goksel O. (2016). Automatic Measurement of Venous Pressure Using B-Mode Ultrasound. IEEE Trans. Biomed. Eng..

[59] Malagutti1 N., Dehghani A., Kennedy R.A. (2013). Robust control design for automatic regulation of blood pressure. IET Control Theory Appl..

[60] Jovanović S., Jovanović M., Škorić T., Jokić S., Milovanović B., Katzis K., Bajić D. (2019). A Mobile Crowd Sensing Application for Hypertensive Patients. Sensors.

[61] Lee S., Chang J.H. (2016). Oscillometric Blood Pressure Estimation Based on Deep Learning. IEEE Trans. Ind. Inform..

[62] De Lucena V.F., Chaves Filho J.E., Viana N.S., Maia O.B. (2009). A home automation proposal built on the Ginga digital TV middleware and the OSGi framework. IEEE Trans. Consum. Electron..

[63] De Lucena V.F., Viana N.S., Maia O.B., Chaves Filho J.E., Da Silva W.S. (2012). Designing an extension API for bridging Ginga iDTV applications and home services. IEEE Trans. Consum. Electron..

[64] Maglogiannis I., Spyroglou G., Panagopoulos C., Mazonaki M., Tsanakas P. Mobile reminder system for furthering patient adherence utilizing commodity smartwatch and Android devices. Proceedings of the 4th International Conference on Wireless Mobile Communication and Healthcare—Transforming Healthcare Through Innovations in Mobile and Wireless Technologies (MOBIHEALTH).

[65] Breiman L., Friedman J., Stone C.J., Olshen R.A. (1984). Classification and Regression Trees.

[66] Cramér H. (1999). Mathematical Methods of Statistics (PMS-9).

[67] Gomes C.E.M., Lucena V.F., Yazdi F., Göhner P. An intelligent medicine cabinet proposed to increase medication adherence. Proceedings of the IEEE 15th International Conference on e-Health Networking, Applications and Services (Healthcom 2013).

[68] Kasinski A., Schmidt A., Kurzynski M., Puchala E., Wozniak M., Zolnierek A. (2007). The Architecture of the Face and Eyes Detection System Based on Cascade Classifiers. Computer Recognition Systems 2.

[69] Castrillón-Santana M., Deniz O., Antón-Canalís L., Lorenzo-Navarro J. Face and facial feature detection evaluation: Performance evaluation of public domain haar detectors for face and facial feature detection. Proceedings of the III International Conference on Computer Vision Theory and Applications (VISAPP’2008).

[70] Altman N.S. (1992). An Introduction to Kernel and Nearest-Neighbor Nonparametric Regression. Am. Stat..

[71] Lu G.F., Zou J., Wang Y. (2012). Incremental complete LDA for face recognition. Pattern Recognit..

[72] Santana S.S.S. (2016). Reconhecimento Facial Utilizando Lda Para Auxílio Ao Controle De Acesso A um Armário de Medicamentos Inteligente.

[73] Singh N., Varshney U. Patterns of effective medication adherence: The role of wireless interventions. Proceedings of the 2014 Wireless Telecommunications Symposium.

[74] He S., Chan S.-G. (2016). Wi-Fi Fingerprint-Based Indoor Positioning: Recent Advances and Comparisons. IEEE Commun. Surv. Tutor..

[75] Yang C., Shao H. (2015). WiFi-based indoor positioning. IEEE Commun. Mag..

[76] Li W.W., Iltis R.A., Win M.Z. A smartphone localization algorithm using RSSI and inertial sensor measurement fusion. Proceedings of the 2013 IEEE Global Communications Conference (GLOBECOM).

[77] Simoes W.C.S.S., Silva L.M., Silva V.J., Lucena V.F. A Guidance System for Blind and Visually, solved.Impaired People via Hybrid Data Fusion. Proceedings of the 2018 IEEE Symposium on Computers and Communications (ISCC).

[78] Simões W.C.S.S., Silva Y.M.L.R., Lucena V.F. (2017). A Location Technique Based on Hybrid Data Fusion used to Increase the Indoor Location Accuracy. Procedia Comput. Sci..

[79] Silva Y.M.L.R., Simões W.C.S.S., Naves E.L.M., Bastos Filho T.F., Lucena V.F. (2018). Teleoperation Training Environment for New Users of Electric Powered Wheelchairs Based on Multiple Driving Methods. IEEE Access.

[80] Dima A.L., Dediu D. (2017). Computation of adherence to medication and visualization of medication histories in R with AdhereR: Towards transparent and reproducible use of electronic healthcare data. PLoS ONE.

[81] Matthews B.W. (1975). Comparison of the predicted and observed secondary structure of T4 phage lysozyme. Biochim. Biophys. Acta (BBA)-Protein Struct..

[82] Zhu W., Zeng N., Wang N. (2010). Sensitivity, specificity, accuracy, associated confidence interval and ROC analysis with practical SAS implementations. NESUG Proc. Health Care Life Sci. Baltim. Md..

[83] Park S.H., Goo J.M., Jo C.H. (2004). Receiver operating characteristic (ROC) curve: Practical review for radiologists. Korean J. Radiol..

[84] Kuncheva L.I. (2004). Combining Pattern Classifiers: Methods and Algorithms.

[85] Kittler J., Hatef M., Duin R.P.W., Matas J. (1998). On combining classifiers. IEEE Trans. Pattern Anal. Mach. Intell..

